# Sterile inflammation as a factor in human male infertility: Involvement of Toll like receptor 2, biglycan and peritubular cells

**DOI:** 10.1038/srep37128

**Published:** 2016-11-16

**Authors:** C. Mayer, M. Adam, L. Glashauser, K. Dietrich, J.U. Schwarzer, F.-M. Köhn, L. Strauss, H. Welter, M. Poutanen, A. Mayerhofer

**Affiliations:** 1 Biomedical Center (BMC), Cell Biology, Anatomy III, Ludwig-Maximilians-Universität (LMU), D-82152 Planegg, Germany; 2 Turku Center for Disease Modeling and Department of Physiology, Institute of Biomedicine, University of Turku, FL-20520 Turku, Finland; 3 Andrology-Center, D-81241 Munich, Germany; 4 Andrologicum, D-80331 Munich, Germany

## Abstract

Changes in the wall of seminiferous tubules in men with impaired spermatogenesis imply sterile inflammation of the testis. We tested the hypothesis that the cells forming the wall of seminiferous tubules, human testicular peritubular cells (HTPCs), orchestrate inflammatory events and that Toll like receptors (TLRs) and danger signals from the extracellular matrix (ECM) of this wall are involved. In cultured HTPCs we detected TLRs, including TLR2. A TLR-2 ligand (PAM) augmented interleukin 6 (IL-6), monocyte chemo-attractant protein-1 (MCP-1) and pentraxin 3 (PTX3) in HTPCs. The ECM-derived proteoglycan biglycan (BGN) is secreted by HTPCs and may be a TLR2-ligand at HTPCs. In support, recombinant human BGN increased PTX3, MCP-1 and IL-6 in HTPCs. Variable endogenous BGN levels in HTPCs derived from different men and differences in BGN levels in the tubular wall in infertile men were observed. In testes of a systemic mouse model for male infertility, testicular sterile inflammation and elevated estradiol (E2) levels, BGN was also elevated. Hence we studied the role of E2 in HTPCs and observed that E2 elevated the levels of BGN. The anti-estrogen ICI 182,780 blocked this action. We conclude that TLR2 and BGN contribute to sterile inflammation and infertility in man.

Signs of sterile inflammation of the testes often accompany impaired spermatogenesis in men. Increased numbers of immune cells (mast cells and macrophages) accumulate specifically in the wall of seminiferous tubules, which frequently show a fibrotic change of their architecture^1^,^2^,^3^,^4^. Testicular mast cells and macrophages secrete, among others, TNFalpha, which can target the cells of the tubular wall (testicular peritubular cells; TPCs) and stimulate the production of IL-6 and MCP-1 upon activation of TNFalpha receptors. This may be a means of promoting inflammation and infertility in man^5^ and point to an important role of TPCs.

Some of the changes typical for male infertility patients are also found in a transgenic mouse expressing human aromatase (AROM+^6^,^7^,^8^,^9^,^10^). These mice have increased levels of testicular estradiol (E2) and after 4 months of age, show signs of deteriorating spermatogenesis and sterile inflammation, characterized by interstitial and peritubular fibrosis, increased presence of immune cells and elevated testicular levels of TNFalpha. With respect to human infertility, at least some men with impaired sperm production also have an altered testosterone (T)/E2 ratio^11^, hinting to a role of E2 in this process.

Recent studies of isolated human TPCs (HTPCs) revealed that they secrete a plethora of factors, including components of the ECM, growth factors and immunological factors^12^,^13^. For example, decorin (DCN) and biglycan (BGN) are closely related members of the family of small leucine-rich proteoglycans (SLRP), abundantly produced by HTPCs *in vivo* and *in vitro*^14^. Besides serving as structural ECM proteins, they may have additional distinct roles. DCN, for example, can interfere with several growth factors and their signaling. Hence it is possible that the observed high decorin levels in fibrotic tubular walls of the testes of infertility patients may contribute to male infertility in this way^15^,^16^. Studies in nonhuman primate testes further indicate that testicular levels of DCN are inversely correlated to the sexual maturity^16^. BGN secreted by HTPCs was recently identified as a ligand of the spermatogonial stem cell receptor FGFR3c^17^. In addition a series of studies provide compelling evidence that it can also act as a ligand at Toll like receptors (TLRs), namely TLR2/4, expressed by macrophages. Hence, BGN may be an ECM-derived factor able to foster inflammation^18^,^19^,^20^,^21^.

Whether HTPCs express TLRs, in particular TLR2, and whether BGN serves as a ligand and may be able to influence HTPCs is not known. We therefore studied the expression of TLRs and BGN, examined aspects of BGN regulation and the possibility that it can function as a signal molecule from the ECM to foster inflammation in human testes. We employed HTPCs as a cellular model and studied testicular samples of infertility patients and AROM+ mice.

## Results

### Expression of TLRs by HTPCs and functionality of TLR2

**Figure 1:**
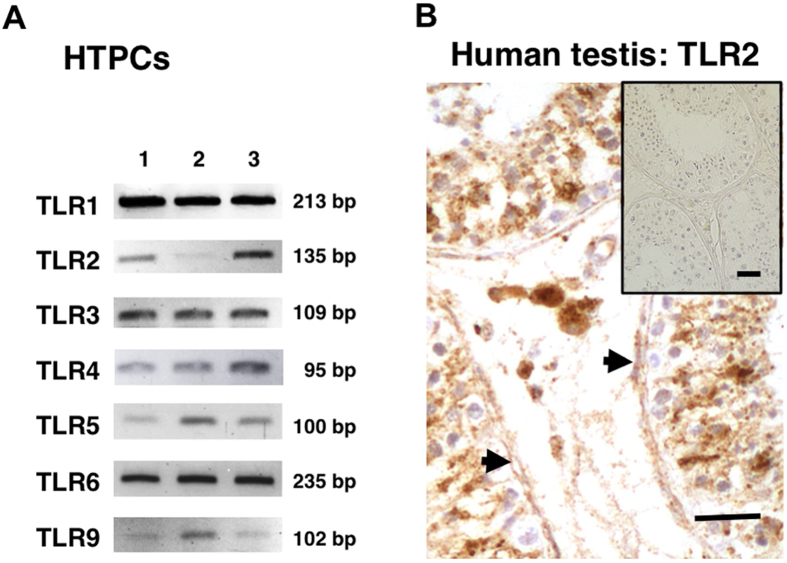
TLR2 in cultured HTPCs and human testis. (**A**) RT-PCR studies showed the presence of TLRs in HTPCs derived from 3 individuals. Note that gels displayed were cropped from different experiments (see Supplementary Information for original gels). (**B**) Immunohistochemical identification of TLR2 in a section of a human testis. Note immunoreactive peritubular cells (arrows). In addition, cells within the tubules, presumably Sertoli cells, and interstitial cells were stained. Insert: result of control experiment (omission of antibody); Bar = 20 μm.

**Figure 2:**
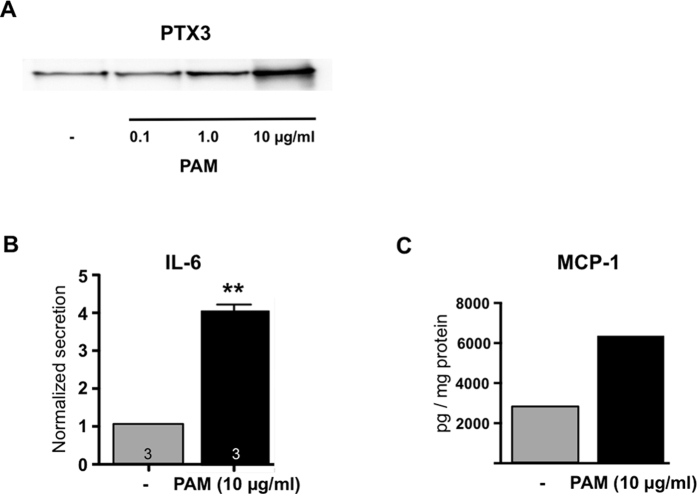
TLR2 in cultured HTPCs are functional. (**A**–**C**) PAM was used to study functionality of TLR2. (**A**) It increased the cellular levels of PTX3 (result of one of n = 3 Western blot experiments is shown; note that the blot displayed was cropped; see Supplementary Information for the original blot). (**B**,**C**) PAM increased the secretion of IL-6 (n = 3) and MCP1 (experiment using HTPCs from a single patient). Results shown were obtained after 24 h using up to 10 μg/ml PAM. **p < 0.01.

A screening of individual (n = 3) HTPC-samples by RT-PCR, followed by sequencing revealed that most TLRs were detectable, including TLR2 (Fig. 1A). TLR7, TLR8 and TLR10 were not detectable in the HTPC-samples examined. Using a commercial antibody, TLR2 protein was identified in human testicular sections in typical peritubular cells, as well as in cells of the tubular and interstitial compartments (Fig. 1B). PAM (Pam3Cys-Ser-(Lys)4), a ligand for TLR2/1^22^,^23^, evoked strong responses in HTPCs. It augmented the production of PTX3 (Western blotting) and the secretion of IL-6 and MCP-1 (ELISA; Fig. 2). This indicates that the TLR2 is functional in HTPCs.

### Role of BGN and expression of BGN by HTPCs and *in vivo*

**Figure 3:**
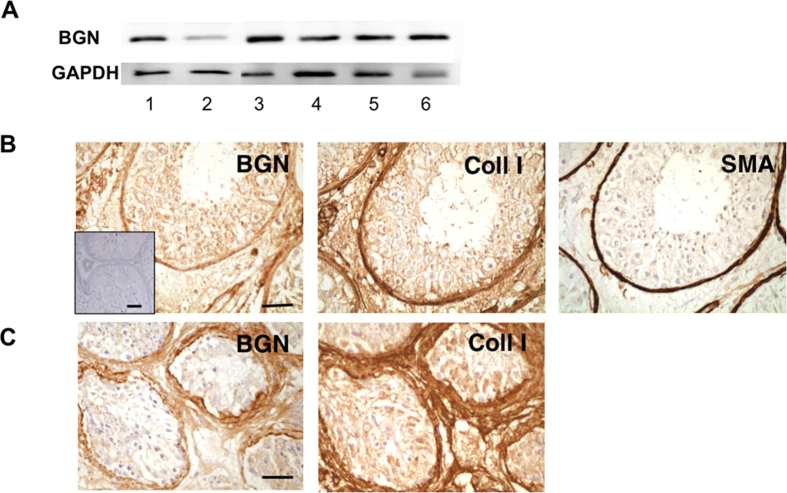
BGN in HTPCs and the human testis. (**A**) BGN protein levels in human cultured HTPCs derived from six different patients (1–6) by Western blotting revealing a distinctive band of expected size at 42 kDa. Note that the levels of BGN protein vary. Detection of GAPDH was used to control for loading equal amounts. Note that the blots displayed were cropped; see Supplementary Information for the original blots. (**B**) Immunohistochemical staining of consecutive testicular sections from a patient showing normal spermatogenesis reveal homogenous BGN staining primarily in the tubular wall, in peritubular cells and its ECM. All controls, including pre-adsorption of the antibody with human recombinant BGN (insert) support the specificity of the reaction. Consecutive sections show collagen type 1 and intracellular SMA, indicating that smooth-muscle-like peritubular cells are the producers of BGN. Bar = 50 μm. (**C**) In a sample from a patient with mixed atrophy (MA) syndrome increased levels of BGN are seen in the walls of seminiferous tubules with impairments of spermatogenesis. Note in the consecutive section that collagen type 1 accumulates in the same areas. Bar = 50 μm.

When HTPCs derived from different men were studied, we observed that their cellular contents and thus production of BGN varied (Fig. 3A). To examine whether BGN levels also vary *in vivo*, we used immunohistochemistry for testicular samples from normal and infertility patients, namely mixed atrophy patients, in which normal and impaired spermatogenesis co-exist. The results revealed homogeneous staining for BGN in peritubular cells and the ECM around tubules with ongoing spermatogenesis, a result in line with previous studies^14^. In consecutive sections, immunoreactive smooth-muscle actin (SMA)-positive peritubular cells and collagen type I in the tubular wall, showed that BGN is a protein associated with this testicular compartment (Fig. 3B). In the wall of tubules with impaired spermatogenesis, the pattern of BGN was altered. Areas of increased BGN-staining of the tubular wall were seen, as well as increased levels of collagen type 1, implying increased production and accumulation in the altered ECM (Fig. 3C), indicative of fibrotic remodeling. All controls, including pre-adsorption of the BGN-antiserum with human recombinant BGN were negative (Fig. 3B, insert).

**Figure 4:**
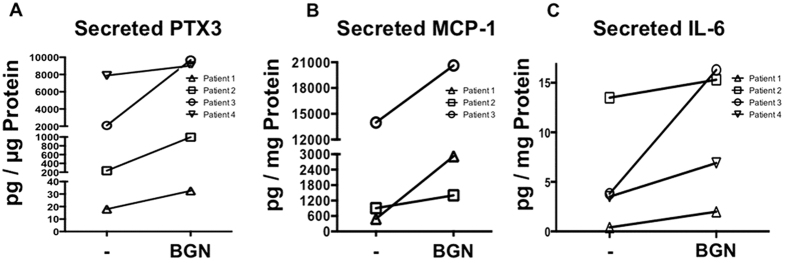
BGN actions on HTPCs. (**A**–**C**) ELISA measurements of secreted (**A**) PTX3 (n = 4), (**B**) MCP-1 (n = 3) and (**C**) IL-6 (n = 4) from individual patients in response to stimulation with BGN (10 μg/ml for 24 h) are shown. Note robust increases but strong inter-individual differences in basal levels and in the degree of the increments.

We next tested whether BGN^13^ is able to activate TLR2^18^,^19^,^20^ and examined the actions of recombinant human BGN in HTPCs. ELISA measurements showed that it specifically increased PTX3-, MCP-1- and IL-6-secretion. The augmenting actions were seen in all human samples derived from individual patients, yet both basal levels and the degree of the increments varied substantially (Fig. 4). When BGN was boiled for 10 min its ability to increase IL-6 was abolished. (BGN induced a 4.2 fold elevation of IL-6 levels over basal levels, which was abolished when BGN was used, which had been heat-inactivated by boiling).

### TLR2 and BGN in AROM+

**Figure 5:**
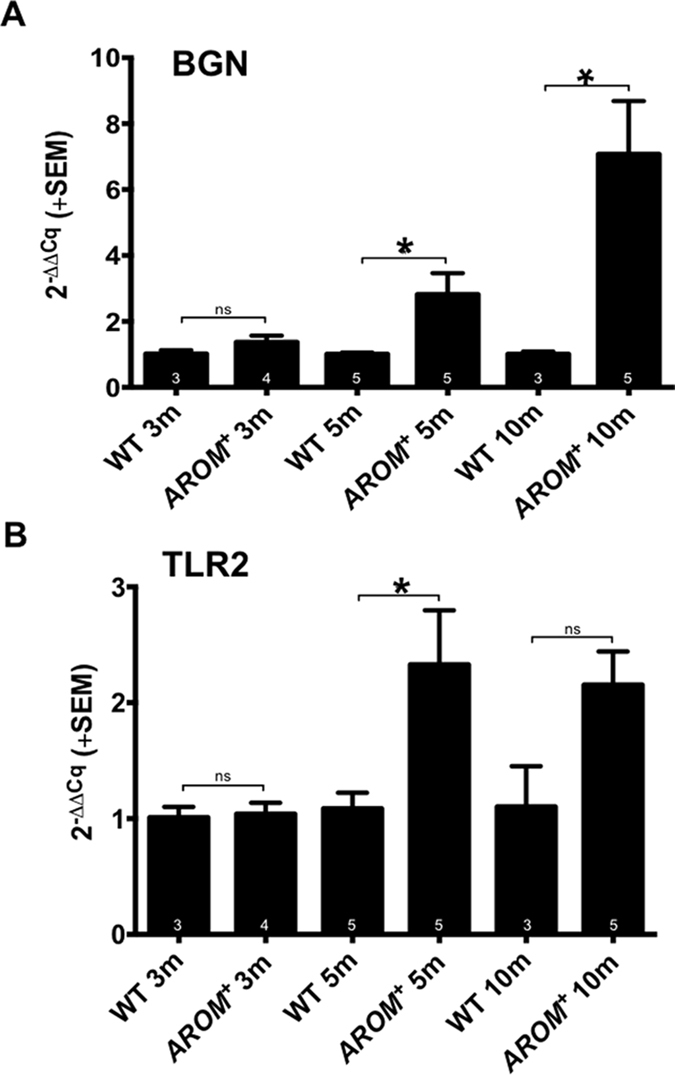
Age-dependent changes in mRNA expression of BGN and TLR2 in wild type and AROM+ mice. Levels of (**A**) BGN and (**B**) TLR2 mRNA in testes of wild type mice (WT) and aromatase transgenic mice (AROM+) at 3, 5 and 10 months (m) are shown. Data were normalized to L19 levels of the corresponding age-matched wild type mouse, which was equalized to 1. Results are the mean + SEM; n as indicated in the columns; *p < 0.05; ns: not statistically significant.

In testes of AROM+ mice qRT-PCR studies revealed significantly increased levels of testicular BGN and TLR2 (Fig. 5) starting at 5 months of age. AROM+ represent a mouse model for sterile inflammation and infertility with elevated testicular E2 and TNFalpha levels^6^,^7^. This led us to explore the roles of E2 and TNFalpha in HTPCs.

### Regulation of BGN and TLR2 in HTPCs

**Figure 6:**
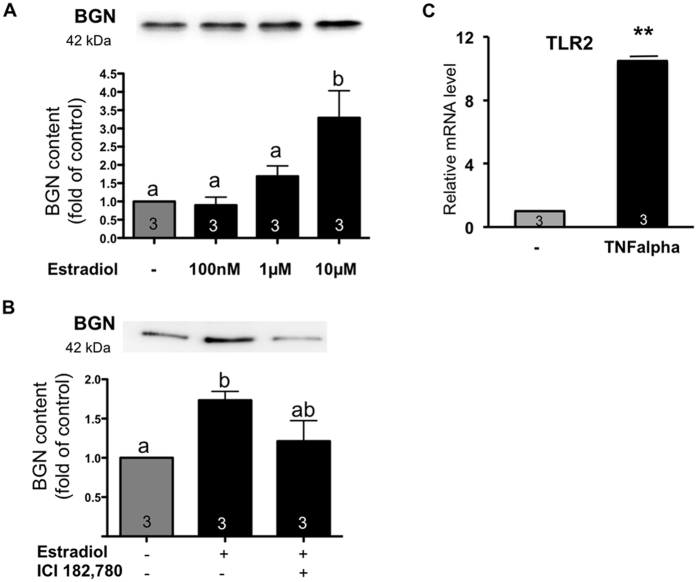
Regulation of BGN by E2, effect of ICI 182,780 on E2 action in cultured HTPCs and regulation of TLR2 by TNFalpha. (**A**) E2 treatment (100 μM, 1 μM, 10 μM) for 3 days led to a significant increase in BGN protein levels. Note that the blots displayed were cropped; see Supplementary Information for the original blots. (**B**) The blocker ICI 182,780 prevented the increase of BGN protein via E2 after 7 days (right). Examples of Western blot bands are depicted above. They were normalized to control values, as shown below. Results are given in arbitrary units (a.u.); mean + SEM (n = 3). Different letters indicate statistically significant differences between the groups (p < 0.01). Note that the blot displayed was cropped; see Supplementary Information for the original blot. (**C**) As revealed by qPCR studies, stimulation of HTPCs with TNFalpha for 24 h provoked a 10fold increase (**p < 0.01) of TLR2 mRNA. The mRNA data are depicted after normalization to L19 and the control was equalized to 1. Results are the mean + SEM (n = 3).

In HTPCs, we found that E2 in a concentration-dependent manner increased the protein levels of BGN and the anti-estrogen ICI 182,780 blocked the E2 action (Fig. 6A,B). TLR2 mRNA levels increased upon treatment of HTPCs with TNFalpha (Fig. 6C).

## Discussion

TLRs play a crucial role in the innate immune response by sensing microorganisms and endogenous danger molecules^24^,^25^. The presence of TLR mRNAs in human testes was shown previously^26^, but specifically the expression of TLRs by HTPCs has not been investigated before. Our RT-PCR results indicate that 7 of the 10 members of the human TLR family can be detected in HTPCs. Notably, TLR7, TLR8 and TLR10 were not detected. Corresponding TLR proteins in human testicular samples could not be fully explored, as with one exception commercial antibodies tested proved not to be suitable for immunohistochemistry in the fixed paraffin-embedded testicular material available. A TLR2 antibody yielded however results in immunohistochemistry of human testicular samples, which support the expression of this receptor subtype among others in peritubular cells.

We focused our study on TLR2 of HTPCs, which is important for the recognition of pathogen-associated microbial patterns and other danger signals. To test its functionality we used PAM, a ligand at TLR2^22^,^23^. We observed a concentration-dependent increase in PTX3-levels of HTPCs. The levels of IL-6 and MCP-1 were increased by PAM, as well. Due to the fact that human testes, human Sertoli cells and spermatogonia are not available for mechanistic studies, the roles of these potent, in general immunologically active molecules in the human testis cannot be studied directly and must be deduced from cellular studies and animal models.

PTX3 has only recently been identified by own work as a major product of HTPCs^13^ and its role in the testis in general remains unknown. Based on present knowledge, it may serve as a key factor of humoral innate immunity^27^,^28^,^29^,^30^,^31^. New information indicates that it serves as an endogenous adjuvant for B-cells, at least in spleen^32^. PTX3 is being discussed as a potential biomarker for infectious diseases, as it is detectable in the circulation^29^,^30^. This raises the possibility that it may qualify also as a biomarker for male infertility. Depending on the prevailing pH, PTX3 may also foster fibrosis^33^. Fibrosis is a complex process involving cell proliferation and activation. In HTPCs, neither PAM nor BGN, which increase PTX3, led to changes in cell number/viability, as concluded from ATP measurements (Supplementary Figure 1). Whether this function of PTX3 therefore is of importance in the human testis, where fibrotic remodeling of the tubular wall typically occurs in infertility, remains to be shown.

MCP-1 was described previously as a factor released by HTPCs^5^, and acts as a chemo-attractant factor for macrophages/monocytes, which are significantly augmented in the testis of infertility patients^2^.

IL-6 is a prototype pro-inflammatory cytokine^5^ with several possible modes of actions. In animal models recent information obtained in mice shows that it acts via a transcription factor (Zfp637) directly on spermatogonia and interferes with germ cell differentiation^34^. In rodent animal models, IL-6 can also disrupt the blood-testis barrier formed by Sertoli cells, e.g. by inhibiting protein degradation in Sertoli cells^35^. A very recent report studied expression of different tight junction proteins, which form this barrier in human and rodent testes^36^. The authors conclude that molecular components of the blood-testis barrier in the human testis differ significantly from the ones in rats and mice. The authors therefore caution that rodents may not be comparable with humans in this respect. Yet their study identified claudin-11 as a reliable barrier protein in rodents and humans. The authors of the study also reported that in testes of men suffering from various degrees of infertility distinct small changes in the immunoreactive pattern of claudin-11 occur. Our immunolocalization of claudin-11 in our human samples does not rule out small changes (Supplementary Figure 2). However whether this implies leaky tight junctions would require functional studies, which are not possible in man. Nevertheless in order to gain further insights, we studied claudin-11 levels in AROM+ and age matched wild-type mice, at an age when inflammation and impairment of spermatogenesis are in full swing. The results showing unchanged claudin-11 levels (Supplementary Figure 2) would not favor massive alteration. Furthermore, given that the blood-testis barrier may leak or break down, immune cells could gain access to the tubular compartment. This was not seen in our samples of mixed atrophy and normal specimen. We observed CD68-positive macrophages only in the interstitial areas and in the tubular wall (Supplementary Figure 2A/B).

PAM is a ligand at TLR1 and 2, which are both detectable in HTPCs. While the proof of principle-experiment provides strong evidence for the functionality of TLR1/2 expressed by HTPCs, this synthetic triacylated lipopeptide is most likely not the normal ligand of TLRs in peritubular cells *in vivo.* Activation by bacteria is unlikely as bacterial infections of the testis are extremely rare. However, danger signals from the ECM may qualify instead as activators of TLRs^18^. Changes of the ECM are typical in testes of men with impairments of spermatogenesis. Especially fibrotic remodeling of the tubular wall occurs^1^,^2^,^3^ and, as indicated by a recent study, BGN is a major secreted proteoglycan derived from HTPCs^13^,^14^,^17^. Interestingly, some of the CD68-positive macrophages identified in human testes were also positive for BGN, a result in line with rodent data^19^,^21^. Thus, testicular macrophage-derived BGN may be a further player in infertility, as well (Supplementary Figure 2B).

BGN can serve as a ligand of TLR2/4^18^,^20^, but not TLR1. Indeed, when we added human recombinant BGN to HTPCs, we observed a robust answer, namely increased protein expression and secretion of PTX3, MCP-1 and IL-6, i.e. the same factors stimulated by PAM. Thus, while involvement of TLR1 (activated also by PAM) and/or TLR4 (activated also by BGN) cannot be ruled out, the results suggest that primarily TLR2 is responsible for the observed actions.

We noted in the present and in previous studies with HTPCs, which are derived from individual human donors (e.g. ref. 37) that the production of factors varied substantially between individual donor-derived cells. This includes variable basal levels and the degree of the response. Because of this and because the endogenous BGN production by HTPCs also varies substantially, we have not attempted additional studies with different concentrations.

Our data further imply that expression of BGN by the cells of the tubular wall and its accumulation in the ECM of the wall varies, when spermatogenesis is impaired. The reasons for this are not fully known, but our cellular studies indicated that E2 is involved and is able to increase the protein levels of BGN in HTPCs. Regulation of BGN by E2 and progesterone has been reported^38^,^39^. In our study the anti-estrogenic drug ICI 182,780^40^ blocked the E2 action on BGN.

We also tested the actions of TNFalpha and tryptase, i.e. factors secreted by testicular immune cells, which accumulate in the tubular wall in male infertility^5^,^41^. None of them, however, affected BGN levels, but TNFalpha treatment increased the levels of TLR2 in HTPCs. TLR2 levels were not changed when E2 was added, a result in line with a recent study in the human oviduct^42^.

We used high concentrations of E2 in our cellular studies to provoke changes in BGN. While it has not been shown if such high levels exist within the microenvironment of the tubular wall in the human, very high local concentrations appear however possible, as several cell types of the human testis express aromatase^43^,^44^. High intratesticular E2 levels are witnessed by the approximately 75-fold higher levels of E2 in testicular venous blood compared to the general circulation^45^. An association between increased testicular E2 and infertility is furthermore suggested by our AROM+ mouse model^6^,^7^, showing age-dependent development of impaired spermatogenesis in connection with increased E2/T ratio^7^. In man, the evaluation of aromatase (CYP19) expression in the testes of patients with non-obstructive azoospermia had indicated significantly elevated levels^10^. Thus, at least some men with impaired sperm production have an altered T/E2 ratio^11^. In these, a role of E2 in male infertility is suggested by the successful use of aromatase inhibitors for the treatment of male infertility. Blockers of aromatase, possibly interfering with Leydig cell aromatization of T^11^, reduce systemic and presumably also testicular levels of E2. Hence there is evidence, linking E2 to male infertility in mice and men^8^, which however must be further elucidated. Our cellular experimental data obtained in HTPCs support such a role. They point to a role of E2 in the induction of testicular BGN, which may fuel inflammation, and may in part explain the effectiveness of this clinical approach.

In summary, our data in human peritubular cells provide novel insights. They show TLR-expression by HTPCs and suggest that increased secretion of BGN by human peritubular cells in the testis may feed back on peritubular cells via TLR2. This initiates responses leading to PTX3, IL6 and MCP-1 secretion. If this occurs in the human testis *in vivo*, it would foster inflammation and indirect evidence for this possibility was likewise provided. While mechanistic studies cannot be performed in humans, results obtained in a systemic model for male infertility and sterile testicular inflammation implied E2 as a factor. The experimental data then obtained in HTPCs show that E2 enhances production of the ECM proteoglycan BGN, which may drive the inflammatory response. Taken together, the results obtained with HTPCs, human testicular sections and with a mouse model for male infertility pinpoint an unknown chain of events, which may lead to sterile inflammation. Components of this system may be targets for therapeutic approaches in male infertility.

## Materials and Methods

### Cell culture of human testicular peritubular cells (HTPCs) and stimulation

The isolation, culture (explant culture) and characterization of HTPCs were performed as described previously^4^,^41^,^46^,^47^. All experiments were performed with cells from up to 6 different patients with normal spermatogenesis (undergoing reconstructive surgery of the vas deferens, 36–52 years old) using passages 5–12. For the scientific use of cells, the patients had granted written Informed Consent. The local Ethical Committee (Ethikkommission, Technische Universität München, Fakultät für Medizin, München, project number 5158/11) has approved the study. All experiments were performed in accordance with relevant guidelines and regulations (including laboratory and biosafety regulations). Stimulation with TNFalpha (5 ng/ml; Sigma-Aldrich, Taufkirchen, Germany), PAM (Pam3Cys-Ser-(Lys)4; 10 μg/ml; from Biomol GmbH, Hamburg, Germany) as well as human recombinant BGN (10 μg/ml; R&D Systems Minneapolis, USA; mouse-myeloma cell line-derived) were performed for 24 h in serum free media. Estradiol (17-beta estradiol, E2, Sigma Aldrich, Taufkirchen, Germany) was tested in concentrations of 0.1, 1.0 and 10 μM and added for 3–7 days. In some cases, cells were pre-incubated for 30 min with ICI 182,780, 0.5nM; (Tocris, Bristol, UK; Howell *et al*.^40^) before stimulation with 10 μM E2. Ethanol (Merck, Darmstadt, Germany) was used as solvent for E2 and was included as control in these experiments.

### Immunohistochemistry

Testicular biopsies, embedded in paraffin, were identical to the ones described in previous studies and were processed for immunohistochemical studies as mentioned^4^. The local Ethical Committee (Ethikkommission, Technische Universität München, Fakultät für Medizin, München, project number 5158/11) has approved the study. A primary antibody against BGN (1:200; polyclonal affinity isolated rabbit anti-human BGN; #ab49701, Abcam, Cambridge, UK) was applied for Western blotting and immunohistochemistry. A further rabbit antiserum to BGN, used for immunohistochemistry, was bought from Sigma (Deisenhofen; Germany, #HPA003157; 1:500). Rabbit anti-collagen type 1 for immunohistochemistry was from Acris (Herford, Germany #R1038; 1:1000), as was rabbit anti-TLR2 (ARP30551; 1:400). Mouse anti-smooth muscle actin (SMA) for immunohistochemistry stems from from Sigma (AS228; 1:1000). Negative controls included incubation with the corresponding IgG isotype instead of the antiserum, omission of the antiserum, and use of normal serum instead of the antiserum. The BGN antiserum (#ab49701, Abcam) was pre-adsorbed with recombinant BGN and used as a further control for immunohistochemistry. In some cases, sections were counterstained with haematoxylin.

### Western blot and ELISA

Western blots of HTPC lysates were performed as described previously^4^ and results were analyzed as described in ref. 41. The following antibodies were used: mouse anti-human BGN (1:1000; monoclonal, #ab54855, Abcam); rat anti-human PTX3 (1:200; monoclonal, #ab90806, Abcam). Both a mouse anti-human beta-actin (1:10,000; monoclonal, #A5441, Sigma-Aldrich) and mouse anti-human GAPDH (1:10,000; monoclonal, #AKR-001, Cell Biolabs, Inc., San Diego, CA, USA) antibody were used to control for equal loading.

Quantikine ELISA Human CCL2/MCP-1, Quantikine ELISA Human Pentraxin 3/TSG-14 (both from R&D Systems, Minneapolis, USA) and Human IL-6 Platinum Ready-to-use Sandwich ELISA (Affymetrix eBioscience, San Diego, CA, USA) were used according to the instructions of the manufacturers. MCP-1, IL-6 and PTX3 levels were determined in the culture supernatants after BGN treatment (10 μg/ml for 24 h) or a 24 h-treatment with PAM. Results were normalized to protein content of cell homogenates and compared to untreated cells.

### Mouse model

A transgenic mouse line expressing human P450 aromatase under the control of the ubiquitin C promoter (AROM+^6^,^7^) was used. The mice were fed with soy-free natural ingredient food pellets (Special Diets Services, Witham, UK) and tap water ad libitum, and housed in specific pathogen-free conditions at Central Animal Laboratory, University of Turku. The animals were handled under a license by the Finnish Animal Ethics Committee, and by the institutional animal care policies of the University of Turku (Turku, Finland), which fully meet the requirements as defined in the NIH Guide for the care and use of laboratory animals. The protocols for animal experimentations were approved by the Central Animal Laboratory of the University of Turku (licence number: KEK/2011-1503-Strauss). Testes of AROM+ and age-matched wild type (WT) mice were used for qPCR studies.

### RNA isolation of mouse testis and quantitative real-time RT-PCR (qPCR)

Total RNA was isolated from mouse testis using TRIsure reagent according to the manufacturer’s instructions (Bioline, Bioline reagents Ltd., London, UK). One microgram of total RNA was treated with deoxyribonuclease I (DNase I Amplification Grade Kit, Invitrogen Life Technologies, Paisley, UK) and RT-PCR was carried out by using the DyNAmo cDNA synthesis kit (Thermo Scientific, Waltham, MA, USA). The cDNA was diluted 1:50 or 1:100 and quantitative PCR was performed using the DyNAmo Flash SYBR Green qPCR kit (Thermo Scientific Waltham, MA, USA) or the QuantiFast SYBR Green PCR Kit (Qiagen, Hilden, Germany). Samples were analyzed in triplicates. L19 was used as an endogenous control to equalize for the amounts of RNA in each sample, respectively. Mouse qPCR data are depicted as relative to the corresponding age-matched wild type mice. Primer sequences and qPCR conditions are described in Table 1.

### Studies with HTPCs: First strand cDNA synthesis RNA, conventional polymerase chain reaction (PCR) and qPCR

Total RNA from sub-confluent HTPCs was prepared using Qiagen RNeasy Micro-Kit (Qiagen, Hilden, Germany). First strand cDNA synthesis followed by conventional PCR for BGN, TLR110 and L19 was described previously^4^. The identity of the amplicons was confirmed via agarose gel electrophoresis and sequence analysis (GATC, Konstanz, Germany). Primer design (http://primer3.wi.mit.edu) and synthesis (Metabion, Munich, Germany), as well as sequence analysis (GATC), were done as described before^4^. Real time PCR (LightCycler® 96 System, Roche Diagnostics, Penzberg, Germany) using the QuantiFast SYBR Green PCR Kit (Qiagen, Hilden, Germany) was performed as described earlier^4^. Changes in gene expression were normalized to the ribosomal gene L19 and calculated according to the 2-∆∆cq method^48^. Human TLR2 expression levels are depicted relative to untreated controls. Primer sequences and qRT-PCR conditions are described in Table 1. Commercial human spleen cDNA (BD Clontech Inc. Heidelberg, Germany) was used as a positive control.

### Statistics

Results of qPCR studies are expressed as mean + SEM. The effects of different treatments on BGN mRNA and protein expression were analyzed using One Way Analysis of Variance and all pairwise multiple comparison procedures followed by Newman-Keuls test. In the transgenic mouse model unpaired t-tests (two-tailed) were performed to investigate differences in mRNA expression of BGN and TLR2. The effects of TNFalpha treatment on TLR2 mRNA expression of HTPCs were calculated using t-test. Unpaired t-test was employed (Prism, GraphPad Software (version 4.0a), Inc., San Diego, CA, USA) and a probability value of p < 0.05 was considered significant and of p < 0.01 highly significant. For ELISA data, a nonparametric test (Kruskal-Wallis test) was performed.

## Additional Information

**How to cite this article**: Mayer, C. *et al*. Sterile inflammation as a factor in human male infertility: Involvement of Toll like receptor 2, biglycan and peritubular cells. *Sci. Rep.* **6**, 37128; doi: 10.1038/srep37128 (2016).

**Publisher’s note**: Springer Nature remains neutral with regard to jurisdictional claims in published maps and institutional affiliations.

## Supplementary Material

Supplementary Information

## Figures and Tables

**Table 1:** Information about sense (forward) and antisense (reverse) oligonucleotide primers, Genebank ID, amplicon size and annealing temperature used for PCR experiments.

Gene	Species Genbank ID	Sequence (5′-3′)	Amplicon size	Annealing temperature
*BGN*	Human NM_001711.4	Forward - TGG CCT GAA GCT CAA CTA CC Reverse - GCC TGG ATT TTG TTG TGG TC	111 bp	59 °C
*BGN*	Mouse NM_007542.4	Forward - GAC AAC CGT ATC CGC AAA GT Reverse - CAA AGG CTC CTG GTT CAA AG	115 bp	60 °C
*IL6*	Human NM_000600	Forward - AAC CTG AAC CTT CCA AAG ATG Reverse - TCT GGC TTG TTC CTC ACT ACT	159 bp	62 °C
*MCP1*	Human NM_002982	Forward - CAG CCA GAT GCA ATC AAT GCC Reverse - TGG AAT CCT GAA CCC ACT TCT	190 bp	54 °C
*RPL19*	Human NM_000981	Forward - AGG CAC ATG GGC ATA GGT AA Reverse - CCA TGA GAA TCC GCT TGT TT	199 bp	59 °C
*RPL19*	Mouse NM_009078.2	Forward - CTG AAG GTC AAA GGG AAT GTG Reverse - GGA CAG AGT CTT GAT GAT CTC	195 bp	60 °C
*TLR1*	Human NM_003263.3	Forward - GCA TAT TGG GCA CCC CTA CA Reverse - TAG GAA CGT GGA TGA GAC CG	213 bp	60 °C
*TLR2*	Human NM_003264.3	Forward - CTG GAG CCC ATT GAG AAA AA Reverse - CGC AGC TCT CAG ATT TAC CC	135 bp	60/62 °C
*TLR2*	Mouse NM_011905.3	Forward - CTC CCA CTT CAG GCT CTT TG Reverse - TTA TCT TGC GCA GTT TGC AG	110 bp	60 °C
*TLR3*	Human NM_003265.2	Forward - CCC TTT GAT TGC ACG TGT GA Reverse - GAG GTG GAG TGT TGC AAA GG	109 bp	60 °C
*TLR4*	Human NM_138554.4	Forward - AGT CCA TCG TTT GGT TCT GG Reverse - CAA TGG TCA AAT TGC ACA GG	95 bp	60 °C
*TLR4*	Mouse NM_021297.2	Forward - GGA CTG GGT GAG AAA TGA GC Reverse - AGC CTT CCT GGA TGA TGT TG	125 bp	60 °C
*TLR5*	Human NM_003268.5	Forward - AAG CCA GGC CTT CTC TTT GA Reverse - CAG GCC AGC AAA TGT GTT CT	100 bp	60 °C
*TLR6*	Human NM_006068.4	Forward - CCC AAA CGG CAC ATT CTT CC Reverse - TGC CGA GGG GAT GTA GGT TT	235 bp	60 °C
*TLR7*	Human NM_016562.3	Forward - GTG GAA ATT GCC CTC GTT GT Reverse - TGT CAG CGC ATC AAA AGC AT	101 bp	60 °C
*TLR8*	Human NM_016610.3	Forward - GCA GCA ATC GTC GAC TAC AG Reverse - GCT GTA CAT TGG GGT TGT GG	159 bp	60 °C
*TLR9*	Human NM_017442.3	Forward - GTC TTG AAG GCC TGG TGT TG Reverse - AGT TCT CAC TCA GGT CCA GC	102 bp	60 °C
*TLR10*	Human NM_030956.3	Forward - ATG CTA GTT CTG GGG TTG GC Reverse - CCC TGT GCC ATG TTT GTG TG	100 bp	60 °C
