# Supplementary material for: Sterile inflammation as a factor in human male infertility: Involvement of Toll like receptor 2, biglycan and peritubular cells
Source: Sci Rep. 2016 Nov 16;6:37128. doi: 10.1038/srep37128 (PMC5111051; doi:10.1038/srep37128)

Supplementary information:

Sterile inflammation as a factor in human male infertility: Involvement of Toll like receptor 2, biglycan and peritubular cells

C. Mayer[1], M. Adam[1,2], L. Glashauser[1], K. Dietrich[1], J.U. Schwarzer[3], F.-M. Köhn[4], L. Strauss[2], H. Welter[1], M. Poutanen[2], A. Mayerhofer[1*]

[1] Biomedical Center (BMC), Cell Biology, Anatomy III, Ludwig-Maximilians-Universität (LMU), D-82152 Planegg, Germany

[2] Turku Center for Disease Modeling and Department of Physiology, Institute of Biomedicine, University of Turku, FL-20520 Turku, Finland

[3] Andrology-Center, D-81241 Munich, Germany

[4] Andrologicum, D-80331 Munich, Germany

PAM and BGN do not significantly affect ATP levels

Cellular ATP levels of HTPCs, as a measure of cellular viability and cell number, were studied using the CellTiter-Glo Luminescent Cell Viability Assay (Promega, Mannheim, Germany) as described[1]. Unpaired t-test (in case of PAM) or ANOVA (BGN) was employed (Prism, GraphPad Software (version 4.0a), Inc., San Diego, CA, USA) and a probability value of $p < 0.05$ was considered significant. Results of ATP-measurements in HTPCs after 24 h in the absence (control) or presence of PAM or BGN revealed that neither treatment significantly affected ATP-levels (**Supplementary Figure 1).** Individual measurements, means and SD are shown (AU: arbitrary units). Experiments were repeated three times using cells derived from

different patients with comparable results.

Supplementary Figure 1

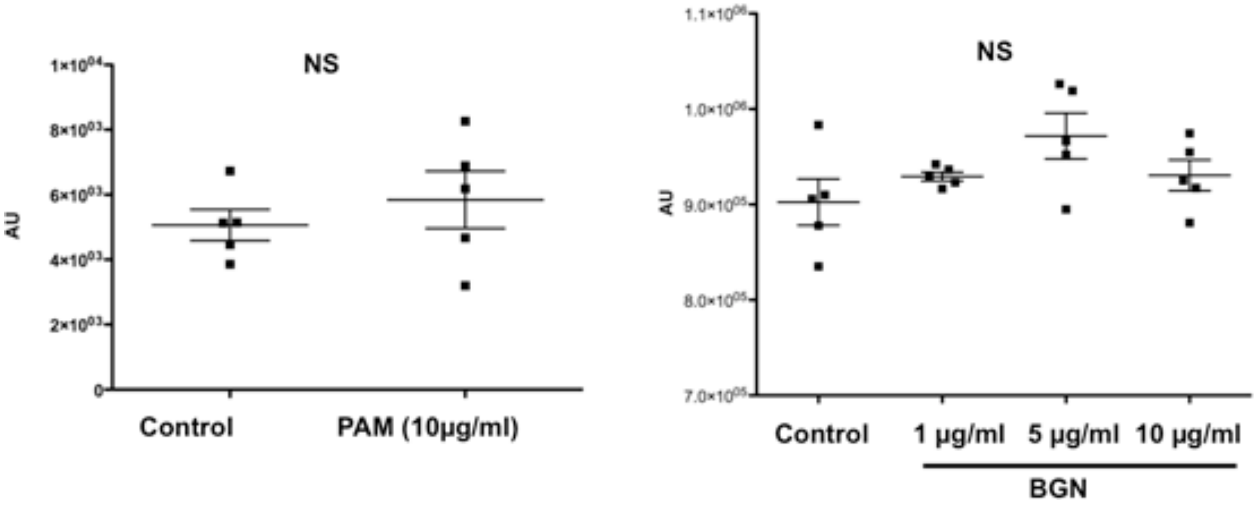

Immunohistochemical detection of claudin-11, CD-68 and BGN in human testes, claudin-11 in WT and AROM+ mouse testes

Additional immunohistochemical studies were performed. The method was performed (n=4 human testicular sections) as described [2,3] using the following commercial antibodies and antisera: mouse anti-CD-68 (1:100, DAKO, Hamburg, Germany); rabbit anti-claudin-11, (#NBP1-82470, 1:100, Novus, Littleton, CO, USA); rabbit anti-BGN (#HPA003157; 1:500, Sigma, Deisenhofen, Germany). As shown in **Supplementary Figure 2A**, immunostaining for claudin-11, a robust marker for Sertoli cell tight junctions, was readily detected, although CD68-positive macrophages were found in the immediate vicinity, as seen in a consecutive section. Some macrophages, expressing CD68 also stained for BGN, as seen in the two

consecutive sections in **Supplementary Figure 2B**. This may indicate that several testicular sources of BGN exist, including macrophages, a result in line with studies in rodents[4,5]. Furthermore, claudin-11 mRNA levels were determined in testes of the 5 month old WT and AROM+ mice, when we observed elevated levels of BGN and TRL2. We used qPCR, as described [2] (see also main text). Primers employed for mouse claudin-11 were as follows: forward primer 5´-TCC TTA TTC TGC TGG CTC TCT-3´ and reverse primer 5´-TCC AAA TGA CTG TGC ATC CC-3´ and L19. Results shown in **Supplementary Figure 2C** revealed that expression levels do not differ (Mann-Whitney test; Prism, GraphPad Software).

Supplementary Figure 2

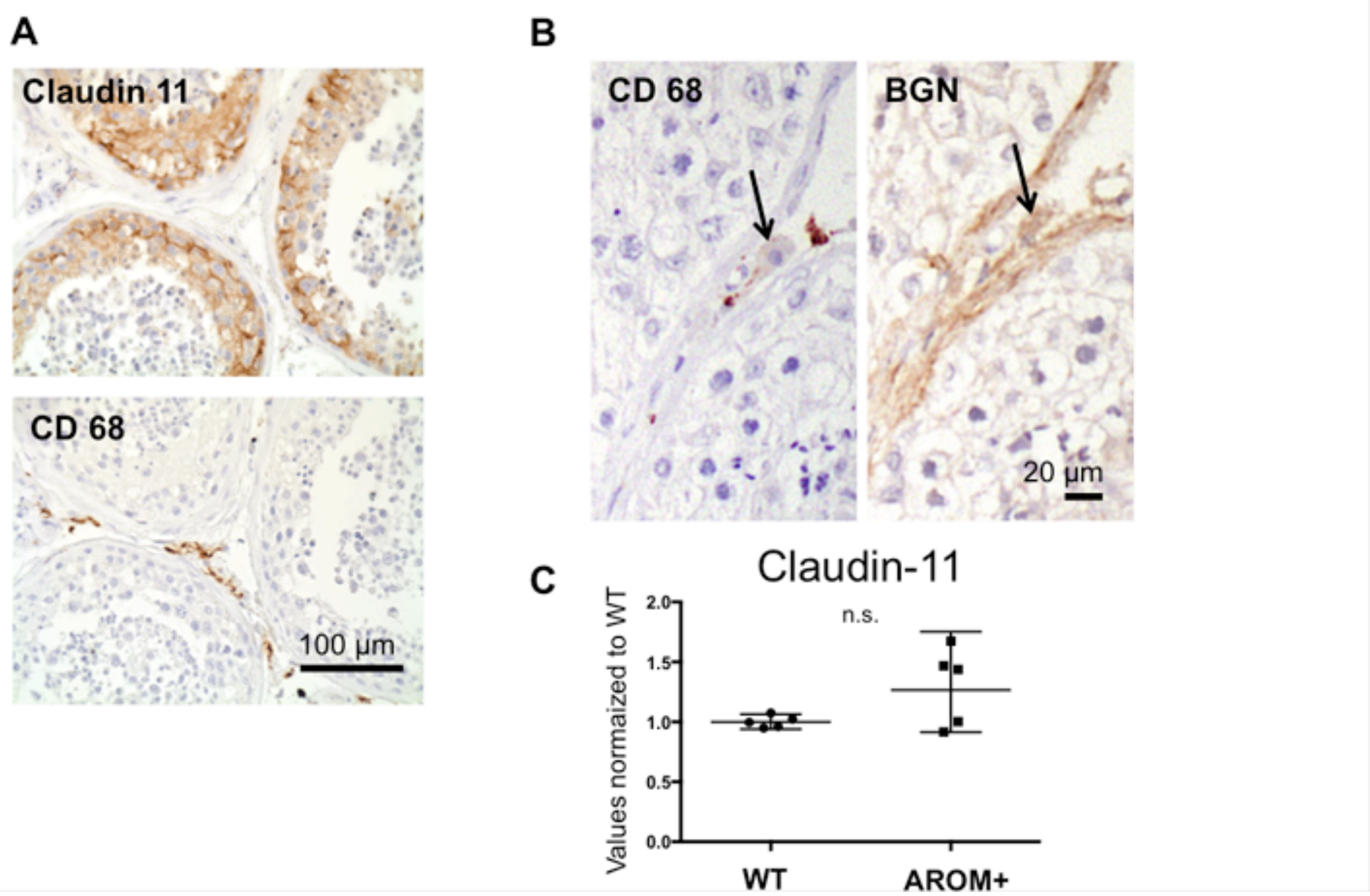

References:

1 Saller, S. *et al.* Dopamine in human follicular fluid is associated with cellular uptake and metabolism-dependent generation of reactive oxygen species in granulosa cells: implications for physiology and pathology. *Human reproduction* **29**, 555-567, doi:10.1093/humrep/det422 (2014).

2 Welter, H. *et al.* Angiotensin II regulates testicular peritubular cell function via AT1 receptor: a specific situation in male infertility. *Molecular and cellular endocrinology* **393**, 171-178, doi:10.1016/j.mce.2014.06.011 (2014).

3 Blohberger, J. *et al.* Readthrough acetylcholinesterase (AChE-R) and regulated necrosis: pharmacological targets for the regulation of ovarian functions? *Cell death & disease* **6**, e1685, doi:10.1038/cddis.2015.51 (2015).

4 Moreth, K., Iozzo, R. V. & Schaefer, L. Small leucine-rich proteoglycans orchestrate receptor crosstalk during inflammation. *Cell cycle* **11**, 2084-2091, doi:10.4161/cc.20316 (2012).

5 Babelova, A. *et al.* Biglycan, a danger signal that activates the NLRP3 inflammasome via toll-like and P2X receptors. *The Journal of biological chemistry* **284**, 24035-24048, doi:10.1074/jbc.M109.014266 (2009).

Additional information: Full length gels and blots. Note that the cropped areas displayed in the composite manuscript figures are marked in red.

Original gels for Figure 1:

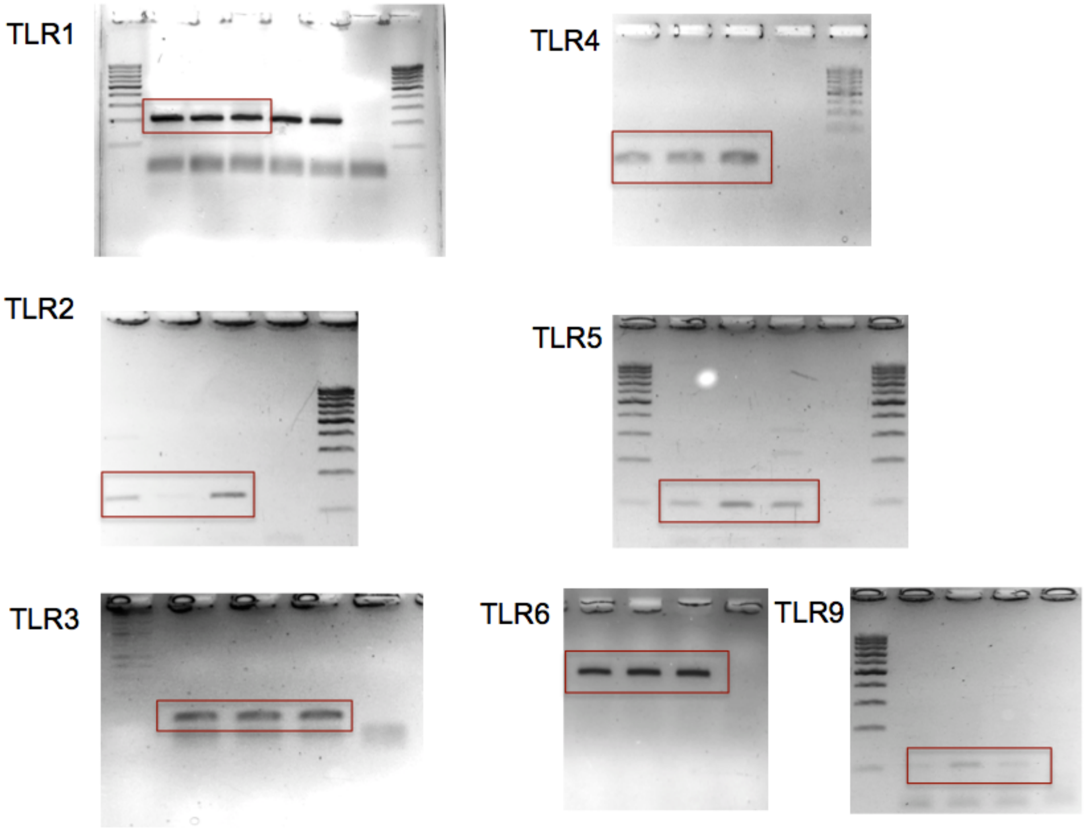

Original Blots for Figures 2 and 3:

Figure 2 (PTX3)

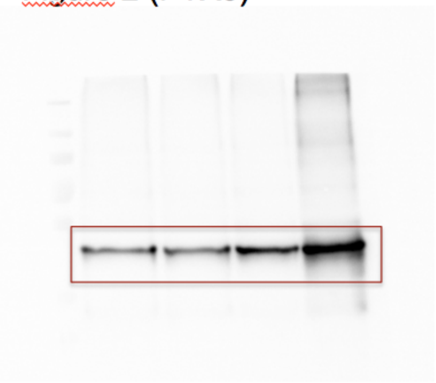

Figure 3 (BGN/GAPDH)

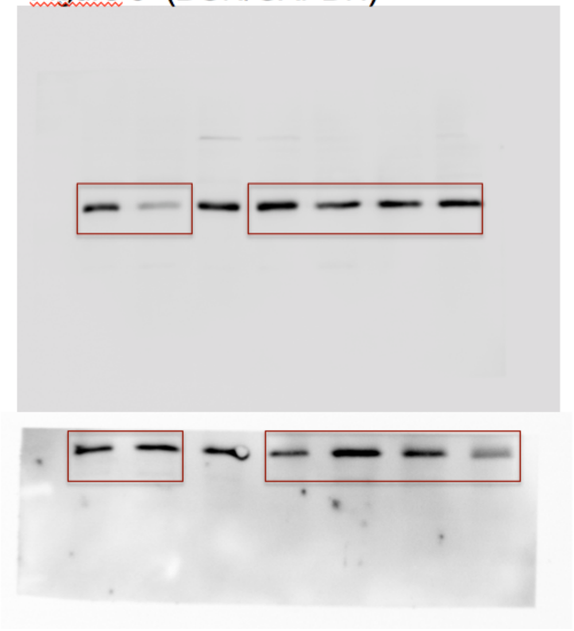

Original Blots for Figure 6:

Figure 6 (BGN)

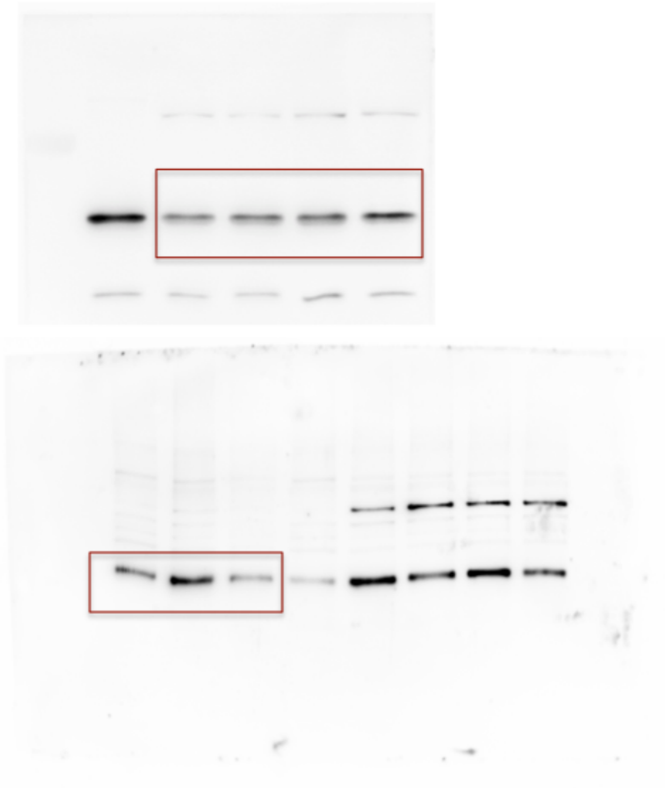

Supplement: Supplementary Information [file srep37128-s1.pdf]
